# Cross-Language Opinion Lexicon Extraction Using Mutual-Reinforcement Label Propagation

**DOI:** 10.1371/journal.pone.0079294

**Published:** 2013-11-15

**Authors:** Zheng Lin, Songbo Tan, Yue Liu, Xueqi Cheng, Xueke Xu

**Affiliations:** Institute of Computing Technology, Chinese Academy of Sciences, Beijing, China; National Institute of Genomic Medicine, Mexico

## Abstract

There is a growing interest in automatically building opinion lexicon from sources such as product reviews. Most of these methods depend on abundant external resources such as WordNet, which limits the applicability of these methods. Unsupervised or semi-supervised learning provides an optional solution to multilingual opinion lexicon extraction. However, the datasets are imbalanced in different languages. For some languages, the high-quality corpora are scarce or hard to obtain, which limits the research progress. To solve the above problems, we explore a mutual-reinforcement label propagation framework. First, for each language, a label propagation algorithm is applied to a word relation graph, and then a bilingual dictionary is used as a bridge to transfer information between two languages. A key advantage of this model is its ability to make two languages learn from each other and boost each other. The experimental results show that the proposed approach outperforms baseline significantly.

## Introduction

Opinion lexicon is a valuable resource for natural language processing and it can be used in sentiment classification [1], sentiment summarization [2], [13], social influence analysis [3] and so on. Although there are several opinion lexicons publicly available, it is hard to maintain a universal opinion lexicon to cover all domains as opinion expressions vary significantly from domain to domain [8]. Hence, mining opinion lexicon for different domains from text corpora automatically has attracted a great deal of attention in the past few years [4]–[8], [10]–[15], [20], [21].

To date, most of the work on opinion lexicon extraction heavily relies on advanced natural language processing tools such as syntactic parsers [5], [8] and information search engine [10] or broad-coverage external resources such as WordNet [11]–[15], [20], [21]. However, these methods are designed to work in a single language and are difficult to generalize to other languages [9], since the resources are imbalanced in different languages. For instance, Qiu et al. [8] propose a bootstrapping method to extract target and opinion word using a dependency parser. However, tools and resources such as dependency parser are available only for a handful of languages, which limits the applicability of these approaches. Hassan and Radev [21] apply a Markov random walk model to a large word graph where the words are connected if they occur in the same WordNet synset, to produce a polarity estimate. However, the dictionary-based methods are unable to find domain dependent sentiment words because most entries in dictionaries are domain-independent. Turney and Littman [10] identify word polarity by looking at its statistical association with a set of positive/negative seed words. One of the limitations of their method is that it requires a large corpus of text to achieve good performance, and this kind of work requires additional access to the Web.

In this paper, we do not aim to beat existing approaches in terms of performance, but take a different perspective and focus on developing a language-independent approach for resource-poor language. Our approach differs from existing approaches in the following three points: first, it does not depend on rich external resources and it is language-independent. Second, our method is domain-specific since the polarity of opinion word is domain-aware. We aim to extract the domain-dependent opinion lexicon (i.e. an opinion lexicon per domain) instead of a universal opinion lexicon. Third, the most importantly, our approach can mine opinion lexicon for a target language by leveraging data and knowledge available in another language. We propose a novel framework to identify the semantic orientationpositive or negative for any opinion word in a bootstrapping [19] way. The basic idea of our approach comes from the mutual reinforcement learning [16]–[18]. The key advantage of this framework is its ability to make two languages learn from each other and boost each other. To the best of our knowledge, multilingual opinion lexicon extraction which combines information of two languages has not been fully investigated.

Our approach propagates information back and forth between source language and target language, which is called mutual-reinforcement label propagation. The mutual-reinforcement label propagation model follows a two-stage framework. At the first stage, for each language, a label propagation algorithm is applied to a large word relation graph to produce a polarity estimate for any given word. This stage solves the problem of external resource dependency, and can be easily transferred to almost any language because all we need are unlabeled data and a couple of seed words. At the second stage, a bilingual dictionary is introduced as a bridge between source and target languages to start a bootstrapping process. Initially, information about the source language can be utilized to improve the polarity assignment in target language. In turn, the updated information of target language can be utilized to improve the polarity assignment in source language as well.

In order to further improve the performance of mutual-reinforcement label propagation, we refine the label propagation algorithm from two aspects: seed word selection and graph construction. For seed word selection, active learning is used in conjunction with semi-supervised learning because it can effectively reduce the demand for labeled samples. To choose the seed words with the maximum coverage, we apply a k-means clustering algorithm to pick unlabeled words to be labeled as seed words by a domain expert. For graph construction, the original graph is replaced by a top-k sub-graph through selecting the top-k related nodes. The top-k sub-graph can ignore those weak word relations to reduce the error propagation.

Our contributions in this study are summarized as follows:

1. We propose a mutual-reinforcement label propagation framework for multilingual opinion lexicon extraction, utilizing information of source language to improve the opinion lexicon extraction of target language, which can overcome the resource-poor problem.
2. To the best of our knowledge, we provide the first attempt to investigate cross-language opinion lexicon extraction and our approach can be generalized to any other languages.
3. We refine the standard label propagation algorithm from two aspects: seed word selection and graph construction, and the experimental results show the effectiveness of our approach.

## Methods

In this section, we describe a method to construct an opinion lexicon through mutual-reinforcement label propagation. In our work, all adjectives in the dataset are regarded as opinion words, and our approach is universal because it can be applied to verbs, adverbs and adjective-noun phrases.

### Mutual-reinforcement Label Propagation Algorithm

**Figure 1:**
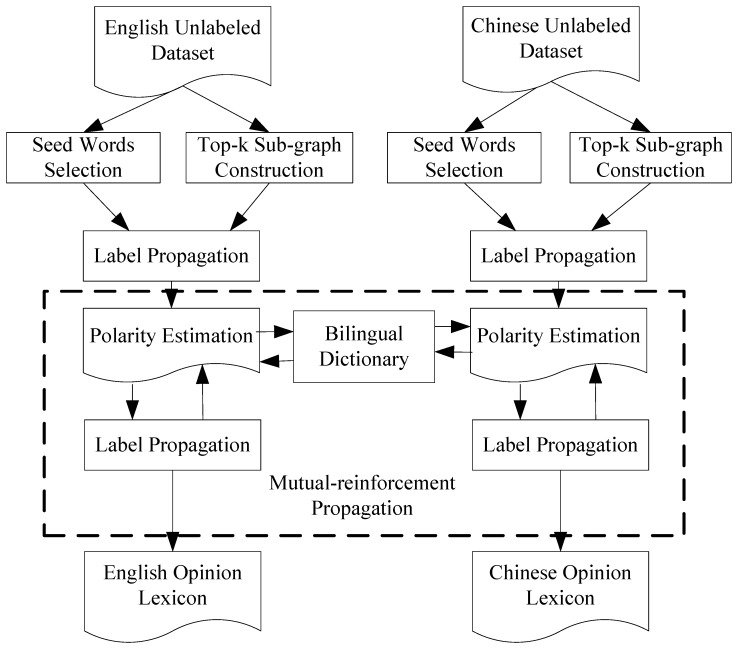
Framework of mutual-reinforcement label propagation.

We aim to have the source language and target language learn from each other and boost each other. An overall framework of our approach is shown in Figure 1. The mutual-reinforcement label propagation model follows a two-stage framework. At the first stage, we apply a label propagation algorithm to a word similarity graph for source and target languages respectively, which can be seen as a process of single propagation. The label propagation algorithm is known to have many desirable properties including convergence and an equivalence to computing random walks through graphs. More specifically, we construct a graph of words where each opinion word denotes a node and two nodes are linked if they are semantically related. The semantic similarity of two nodes is defined by pointwise mutual information (PMI):
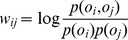
where 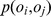 is the probability of word 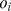 and word 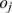 co-occurred in the same sentence, 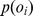 is the probability of word 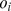 occurred in a sentence and 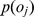 is the probability of word 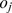 occurred in a sentence.

Let 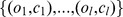 be the labeled nodes, 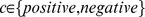, and 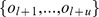 be the unlabeled nodes. Let 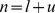. We will use *L* and *U* to denote labeled and unlabeled nodes respectively.

The label propagation algorithm is based on the assumption that nodes connected by an edge with high weight tend to have same label in the graph constructed by labeled data and unlabeled data. It is observed that opinion words in the same sentence have similar sentiment or polarity. Thus, we can assign the same polarity to words with high co-occurrence. We construct a graph where two nodes are linked if they are semantically related, and the polarity estimate problem is formulated as a form of propagation on a graph where a node€s label propagates to neighboring nodes according to their semantic similarity. The polarities are propagated through the edges. Larger edge weights allow polarities to travel through more easily. Define a 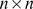 probabilistic transition matrix P.
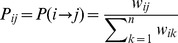
where 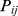 is the probability of transit from node *i* to *j*. Also define a 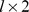 polarity matrix 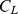, whose *i*th row is an indicator vector for 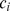, 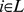. We will compute the polarity matrix *f* for all the nodes. *f* is a 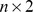 matrix, the rows can be interpreted as the probability distributions over polarities.

We present the label propagation algorithm as follows.

1. Propagate 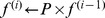.
2. Keep the values of the labeled nodes unchanged 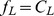.
3. Repeat from step 1 until *f* converges.

In step 1, all nodes propagate their polarities to their neighbors for one step. Step 2 is critical: we want persistent label sources from labeled data. So instead of letting the initial labels fade away, we fix them at 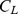. With this constant *push* from labeled nodes, the class boundaries will be pushed through high density regions and settle in low density gaps.

At the second stage, a bilingual dictionary is introduced as a bridge between two languages to start the bootstrapping process. Unlike previous work, the bilingual dictionary is not used to translate the existing opinion lexicon into the target language directly, but seen as a bridge to transfer polarity information back and forth between source language and target language. For example, suppose *s* is a word in source language and its polarity is 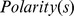, if word *t* is the translation of *s* in the bilingual dictionary, then we think *t* inherits the polarity information of *s* with a certain probability, rather than assigning 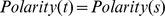 stiffly. In general, the heuristic information of our framework derives not only from the initial seed words but also from the knowledge of another language.

**Figure 2:**
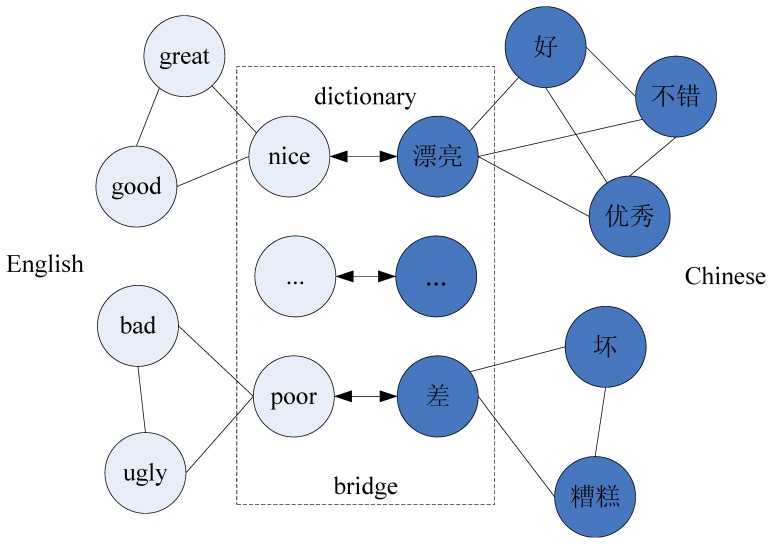
Example of mutual-reinforcement label propagation.

**Figure 3:**
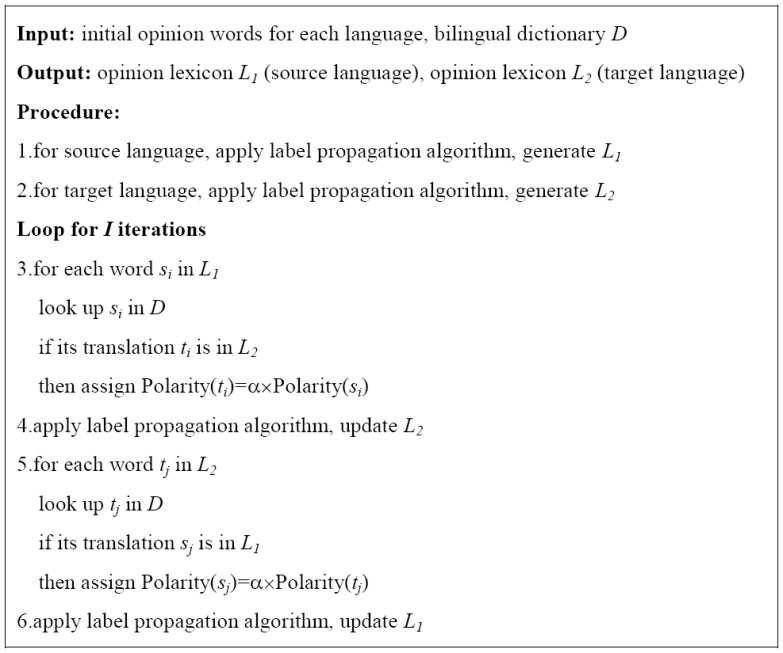
The mutual-reinforcement label propagation algorithm.

For each language, a label propagation algorithm is applied to a large word relation graph, producing a polarity estimate for any given word. In order to establish communication between two languages, a bilingual dictionary is introduced. Figure 2 shows a simple example of mutual-reinforcement label propagation. Suppose that English is a source language and Chinese is a target language. Once the English opinion lexicon is translated into Chinese, we have more polarity information to modify the original Chinese opinion lexicon. For instance, *poor* and is 差 a translation pair in the dictionary. As seen in Figure 2, 坏 is tightly connected with 差. If we know *poor* is a negative word, we may infer that 坏 tends to be a negative word too. In our work, a parameter 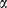 is adopted to prevent the polarity modification from hypercorrection. Conversely, the polarity information of Chinese opinion words can be transferred to English too. Thus, the mutual-reinforcement label propagation algorithm works in a bootstrap mode, by propagating information back and forth between source language and target language. The algorithm of mutual-reinforcement label propagation is shown in Figure 3.

### Seed Word Selection

**Figure 4:**
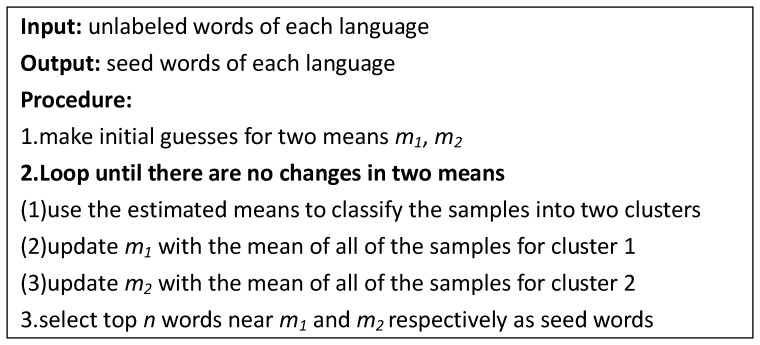
The seed word selection algorithm.

If we have to label a few instances for semi-supervised learning, it may be attractive to let the learning algorithm tell us which instances to label, rather than selecting them randomly [22]. In fact, there is a way that can effectively reduce the demand for labeled data, which is called active learning [23]. In active learning, the most informative data is picked to be labeled by an expert actively. The standard active learning algorithm usually chooses the maximum-entropy data, because the maximum uncertain data contains the maximum information content. However, the maximum-entropy selection strategy is not necessarily able to bring the most significant improvement in some cases. In the process of polarity propagation, we should choose the initial seed words with the maximum coverage instead of the maximum uncertainty, because our goal is to identify the polarity of the unlabeled words by propagating labels of limited seed words. Intuitively, if a labeled word is surrounded by many nodes, it plays an important role in propagating information. Therefore, we think that the words falling into the clustering centers have a crucial effect on the propagation process. In our work, we cluster the opinion words beforehand, and then label the words that are nearest to the clustering centers. In the process of word clustering, the K-means algorithm with two means is applied, and initial clustering centers are selected randomly. The algorithm of seed word selection is shown in Figure 4.

In the seed word selection algorithm, for each opinion word 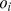, we construct a feature vector 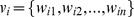, where 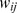 is the PMI value of 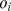 and 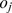, 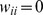. The similarity of two opinion words is measured by the cosine value of the two vectors, which is defined as:
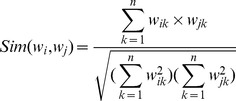



If 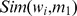 is greater than 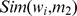), 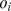 belongs to the first cluster; if not, 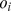 belongs to the second cluster.

### Graph Construction

In graph-based algorithms, it is usually more meaningful to construct a high-quality graph rather than selecting a specific algorithm. There are two major factors which influence the label propagation algorithm: similarity measure function and the number of edges connecting each node. Without loss of generality, the similarity is measured by PMI. In a mutual-reinforcement label propagation model, we use a top-k sub-graph to replace the original graph. In the top-k sub-graph, each node connects to only a few nodes. Such sparse graphs are computationally fast. They also tend to enjoy good empirical performance [22].

The top-k sub-graph is generated from the original graph, which can be divided into three steps. For any given node, the first step is to sort all the edges connecting it according to edge weight. The second step is to reconstruct the graph by retaining the top 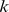 edges for each node. The third step is to normalize the edge weight according to the rule 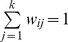.

The advantages of a sparse graph are twofold: first, the number of edges is reduced, which can speed up the algorithm execution and reduce the demand for memory space. Second, the performance of opinion lexicon extraction can be improved because the sparse graph ignores those weak word relations to reduce the error propagation.

## Results

To validate the effectiveness and robustness of the proposed method, we conduct experiments on product reviews of English and Chinese. In order to highlight the domain-specific nature of opinion words, we collect reviews not only from different languages, but also from different domains (electronics, kitchen, network and health). In the process of mutual-reinforcement label propagation, the datasets of the source language and the target language belong to the same domain.

### Experimental Setup

The resources used in the experiments contain unlabeled datasets of two languages and a bilingual dictionary. The bilingual dictionary (http://liuctic.com/zhenglin/) contains 41812 translation pairs. For each language, the datasets cover four domains (Kitchen, Electronics, Health, Network) respectively. The following datasets were collected and used in the experiments:

English Unlabeled Dataset: The English product reviews were collected from Amazon by Blitzer et al. [24] and Li et al. [25]. Each set of the four domains contains 1000 reviews (500 positive and 500 negative reviews).

Chinese Unlabeled Dataset: The Chinese product reviews were collected from www.JD.com. Each set of the four domains contains 10000 reviews (5000 positive and 5000 negative reviews). The size of Chinese unlabeled data is ten times the one of the English datasets, but the quality is much poorer.

Part-of-speech tagging on the English text was performed by Stanford POS tagger (http://nlp.stanford.edu/software/tagger.shtml) and on the Chinese text was performed by ICTCLAS (http://ictclas.org/).

In the mutual-reinforcement label propagation algorithm, the seed word set for each language contains 10 (value of 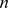 in Figure 4) positive words and 10 negative words, and the parameter 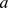 is set to 0.1.

In K-means clustering, for English, the initial two means are *great* and *poor*; for Chinese, the initial two means are 好 (*good*) and 坏 (*bad*).

In the top-k sub-graph construction, the 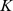 value of English is set to 50; the 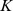 value of Chinese is set to 100.

We selected adjectives with term frequency exceeding a threshold from the corpus to obtain a list of opinion words in each domain. Then, we manually labeled the semantic orientation of every word, and used these labeled word lists as the references in the evaluation. In order to highlight the domain-specific nature of opinion lexicon, we labeled the opinion words according to their domain characteristics. To justify the reliability of this labeling process, we invited three annotators to label the semantic orientation (positive or negative) of the same data. According to the usual practice of voting, the minority is subject to the majority, if there is disagreement about the annotated results. In the experiments, accuracy is used to evaluate the performance of the proposed method, which is measured by counting the number of correctly labeled opinion words.

In order to evaluate the effectiveness of the extracted opinion lexica, we applied them to practical sentiment classification task. We tested the quality of the extracted opinion lexica according to how well they could improve the performance of sentiment classification. The evaluation could provide more objective and reliable judgments and highlight the utility of our approach in practical application. The sentiment classification is unsupervised, thus we only used the labeled dataset for testing and the labeled dataset was not included in the unlabeled dataset. For English sentiment classification, the test set of each domain contains 500 positive reviews and 500 negative reviews. For Chinese sentiment classification, the test set of each domain contains 2000 positive reviews and 2000 negative reviews. There are two evaluation measures in our experiments: polarity assignment accuracy and sentiment classification accuracy. Specifically, polarity assignment accuracy equals to the number of correctly labeled words divided by the number of words labeled; sentiment classification accuracy equals to the number of correctly classified documents divided by the number of documents classified.

We compared our mutual-reinforcement label propagation (MLP) model with the following baseline methods.

LP: The label propagation algorithm was applied to a single language, rather than propagating information back and forth between two languages, which is called single propagation. LP and MLP only differ in whether cross-language relations are used and all the other aspects are identical.

M1: For each domain, we employed the general seed words to start the propagation process.

M2: For each domain, the seed words were selected through picking top n opinion words with high term frequency.

M3: For each domain, the seed words were selected through picking top n opinion words with high PMI value with two polar seed words (*great* and *poor* in English, 好 and 坏 in Chinese).

M4: The label propagation algorithm was conducted on the original graph without top-k sub-graph reduction.

SentiWordNet (http://sentiwordnet.isti.cnr.it/): SentiWordNet is the result of automatically annotating all WORDNET synsets by Baccianella et al. [26].

MPQA (http://mpqa.cs.pitt.edu/): The opinion lexicon was compiled from several sources by Wiebe et al. [27]. Some were culled from manually developed resources. Others were identified automatically using both annotated and unannotated data.

Tsinghua (http://nlp.csai.tsinghua.edu.cn/ 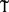
j/sentiment.dict.v1.0.zip): The public opinion lexicon was built by Li and Sun [28].

Hownet (http://www.keenage.com/): The Chinese opinion lexicon is collected from Hownet which is developed for Chinese natural language processing.

Random Walk: The semantic orientation of words is predicted by hitting time in random walks as in the work of Hassan and Radev [21].

### Mutual-reinforcement Label Propagation vs. Baselines

The scale of each opinion lexicon extracted through mutual-reinforcement label propagation is shown in Table 1. The scale of Chinese opinion lexicon is larger than the scale of English opinion lexicon, because the size of Chinese unlabeled data is ten times the one of English dataset.

**Table 1:** Scale of the extracted opinion lexicon.

	English	Chinese
Kitchen	624	1273
Electronics	736	1303
Health	561	1174
Network	659	1186

Tables 2–3 show the accuracy of opinion lexicon extracted by mutual-reinforcement label propagation and the baseline methods. The tables show that our approach performs better than both LP and Random Walk in both languages. We conducted two-paired t-test (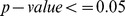) over the results (involving all four domains of two languages together) and found that the improvements were significant over both LP and Random Walk. More specifically, for English, the accuracy of mutual-reinforcement label propagation increases by 0.0328 and 0.0645 respectively compared to the single propagation and random walks on average. As for the English open-source opinion lexica SentiWordNet and MPQA, the accuracy is higher but the coverage is limited. For Chinese, the accuracy of mutual-reinforcement label propagation increases by 0.0269 and 0.0589 respectively compared to the single propagation and random walks on average. As for the Chinese open-source lexica Tsinghua and HowNet, the accuracy is fairly satisfactory because they are built manually, whereas we extract the opinion lexicon from corpora automatically. Since the universal opinion lexicon can not cover many domain-related opinion words and the polarity of opinion word may vary from domain to domain, extracting opinion lexicon from corpora directly is more helpful. From the experimental results in Tables 2–3, we can conclude that the mutual-reinforcement label propagation algorithm outperforms the monolingual polarity prediction algorithm. The better performance can be attributed to the fact that the mutual-reinforcement label propagation algorithm learns more information by combining two languages together. Furthermore, the mutual-reinforcement label propagation algorithm is based on bootstrapping, which enables the source language and target language to learn from each other and boost each other. Note that, in the experiments, the size of English data is much less than the size of Chinese data, thus opinion lexicon extraction for English has great potential for improvement.

**Table 2:** Accuracy of English opinion lexicon acquired by different methods.

Domain	SentiWordNet		MPQA		Random Walk	LP	MLP
	Accuracy	Coverage	Accuracy	Coverage			
Kitchen	0.7926	0.7927	0.9437	0.7120	0.7263	0.7664	0.7944
Electronics	0.7836	0.8116	0.9209	0.7329	0.7317	0.7755	0.8163
Health	0.7805	0.8043	0.9297	0.7132	0.6818	0.6970	0.7475
Network	0.8051	0.8114	0.9194	0.7403	0.7285	0.7561	0.7683
Average	0.7905	0.8050	0.9233	0.7246	0.7171	0.7488	0.7816

**Table 3:** Accuracy of Chinese opinion lexicon acquired by different methods.

Domain	Tsinghua		Hownet		Random Walk	LP	MLP
	Accuracy	Coverage	Accuracy	Coverage			
Kitchen	0.9777	0.6322	0.9523	0.8251	0.7065	0.7500	0.7702
Electronics	0.978	0.6476	0.9524	0.8275	0.7200	0.7470	0.7470
Health	0.9763	0.6147	0.9625	0.8174	0.7075	0.7246	0.7633
Network	0.9763	0.6210	0.9542	0.8172	0.7001	0.7405	0.7892
Average	0.9771	0.6289	0.9553	0.8218	0.7085	0.7405	0.7674

Tables 4–5 show the accuracy of sentiment classification predicted by the extracted opinion lexicon. The t-test (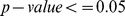) also shows the improvements by our approach are significant over both LP and Random Walk. The improvements show the utility of our approach in practical sentiment classification application. Though the accuracy of open-source opinion lexicon is higher than the one of the automatically extracted opinion lexicon, the sentiment classification based on public opinion lexicon performs worse than our method except HowNet because most of the public opinion lexica suffer from the problems of coverage and domain dependency. Compared to Hownet which is a high-quality public resource maintained by many people, the performance of our method is close to the method based on Hownet. In general, the key advantage of our method is that it provides a cross-language mechanism for opinion lexicon extraction instead of the polarity assignment algorithm itself. The proposed mechanism can be easily combined with a monolingual opinion lexicon extraction algorithm such as an approach based on random walk. Besides, our method is very flexible because it can be generalized to any other language. Essentially, the proposed framework mainly benefits from the mutual-reinforcement principle. By modeling the datasets of two languages together, the mutual-reinforcement label propagation algorithm can learn more knowledge than the monolingual related approaches. An intuitive explanation of the phenomenon is that using more datasets can expand the word co-occurrence, which is helpful to improve the performance of polarity assignment. To the best of our knowledge, the existing approaches for polarity assignment do not incorporate the cross-language mechanism; while our method provides the first attempt to introduce a cross-language mechanism for opinion lexicon extraction.

**Table 4:** Accuracy of unsupervised English sentiment classification based on different methods.

Domain	SentiWordNet	MPQA	RandomWalk	LP	MLP
Kitchen	0.597	0.690	0.595	0.672	0.700
Electronics	0.545	0.620	0.606	0.616	0.657
Health	0.530	0.600	0.508	0.572	0.591
Network	0.531	0.624	0.574	0.630	0.677
Average	0.551	0.634	0.571	0.623	0.656

**Table 5:** Accuracy of unsupervised Chinese sentiment classification based on different methods.

Domain	Tsinghua	Hownet	Random Walk	LP	MLP
Kitchen	0.5655	0.7240	0.6780	0.6400	0.6748
Electronics	0.5840	0.6585	0.6433	0.6445	0.6685
Health	0.5950	0.6375	0.5750	0.5798	0.6238
Network	0.5700	0.6790	0.6423	0.6253	0.6520
Average	0.5786	0.6748	0.6347	0.6224	0.6548

### Influence of Seed Word Selection

Table 6 and table 7 show the average results of different seed word selection methods within the same language. As seen in Table 6 and Table 7, our approach based on K-means clustering outperforms all the other approaches. For English opinion lexicon extraction, we have an improvement of 0.056 over M1, 0.0554 over M2, 0.0623 over M3. For Chinese opinion lexicon extraction, we have an improvement of 0.0264 over M1, 0.0244 over M2, 0.0313 over M3. Except the general seed words of M1, seed words of other methods are all domain dependent. Though M2 and M3 pick seed words for each domain actively, they have no significant advantage over the domain independent method LP. It is because term frequency and PMI strategies are unable to find the seed words with great coverage. However, those seed words with great coverage can be found through K-means clustering. In general, the cluster centers are the densest district of the whole sample space. Besides, K-means clustering guarantees the diversity of the selected seed words because the distance between different cluster centers is large. In summary, the better performance of our approach is due to the fact that the seed words near the cluster centers have great coverage and diversity, which will be helpful to information propagation in a relation graph.

**Table 6:** Accuracy of English opinion lexicon extraction based on different seed word selection methods.

Domain	M1(English)	M2(English)	M3(English)	K-means(English)
Kitchen	0.7383	0.7383	0.7290	0.7664
Electronics	0.6735	0.7143	0.6939	0.7755
Health	0.7374	0.6869	0.6768	0.6970
Network	0.6220	0.6341	0.6463	0.7561
Average	0.6928	0.6934	0.6865	0.7488

**Table 7:** Accuracy of Chinese opinion lexicon extraction based on different seed word selection methods.

Domain	M1(Chinese)	M2(Chinese)	M3(Chinese)	K-means(Chinese)
Kitchen	0.7379	0.7419	0.7218	0.7500
Electronics	0.6798	0.6680	0.6996	0.7470
Health	0.7198	0.7198	0.686	0.7246
Network	0.7189	0.7351	0.7293	0.7405
Average	0.7141	0.7162	0.7092	0.7405

### Original Graph vs. Top-k Sub-graph

**Figure 5:**
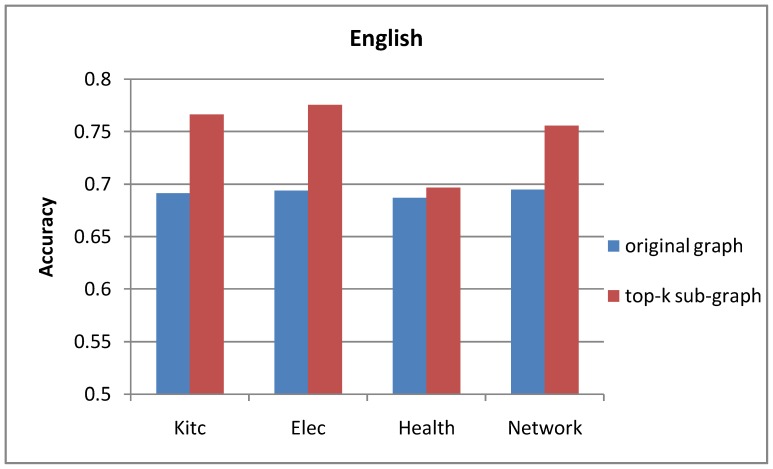
Accuracy of English opinion lexicon extraction based on different graphs.

**Figure 6:**
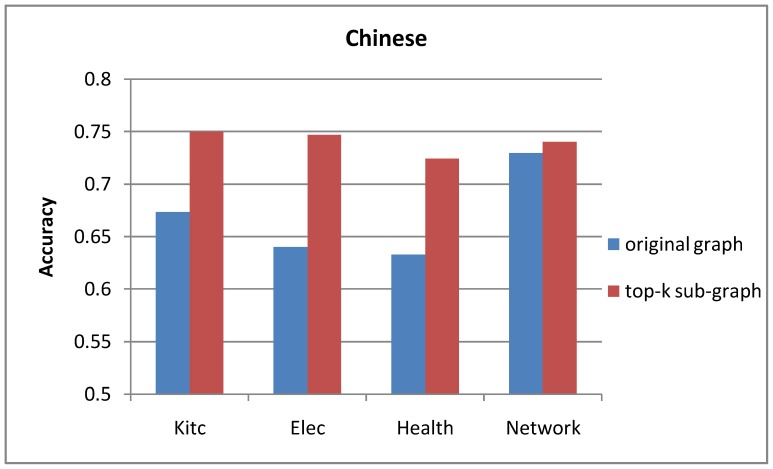
Accuracy of Chinese opinion lexicon extraction based on different graphs.

The comparison results of the opinion lexicon extraction based on different graphs are shown in Figures 5 and 6. From the experimental results in Figures 5 and 6, we can see that the label propagation algorithm based on top-k sub-graph outperforms the standard model based on the original graph in both languages. The results show that our optimization approach is reasonable. By pruning the low-weight edges, the performance of polarity propagation can be improved because some errors can be avoided.

**Figure 7:**
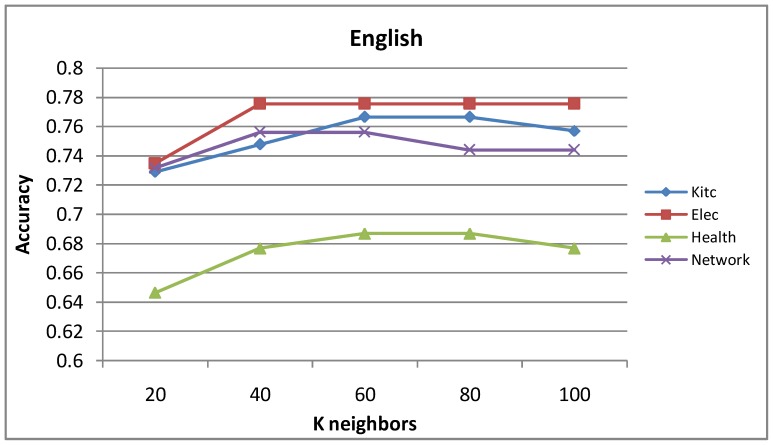
Accuracy of the label propagation algorithm based on top-k sub-graph with different 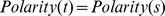 values for English.

**Figure 8:**
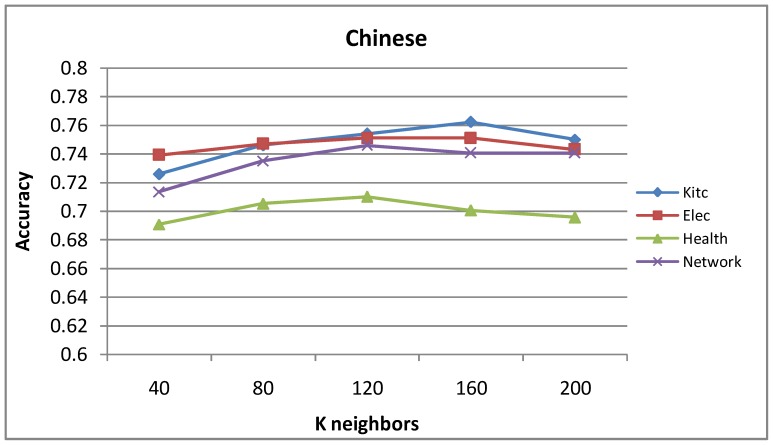
Accuracy of the label propagation algorithm based on top-k sub-graph with different 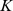 values for Chinese.

Figure 7 and Figure 8 show the influence of parameter 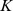 on label propagation algorithm based on top-k sub-graph. We can see that the precision curve rises first and falls later with increasing variable K on four domains of both languages. As seen in Figure 7, when 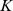 takes the value from 40 to 60, the average accuracy is best for English. As seen in Figure 8, when 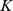 takes the value from 80 to 120, the average accuracy is best for Chinese.

### Influence of Iteration Number

**Figure 9:**
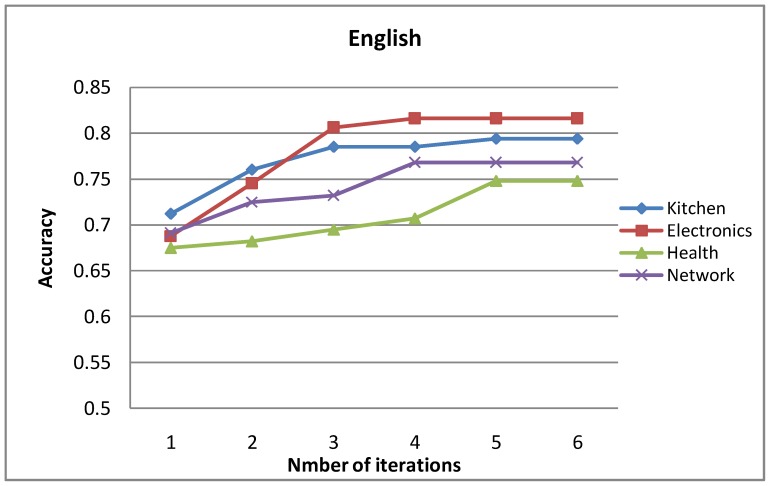
Accuracy of the English opinion lexicon extraction with increasing number of iterations.

**Figure 10:**
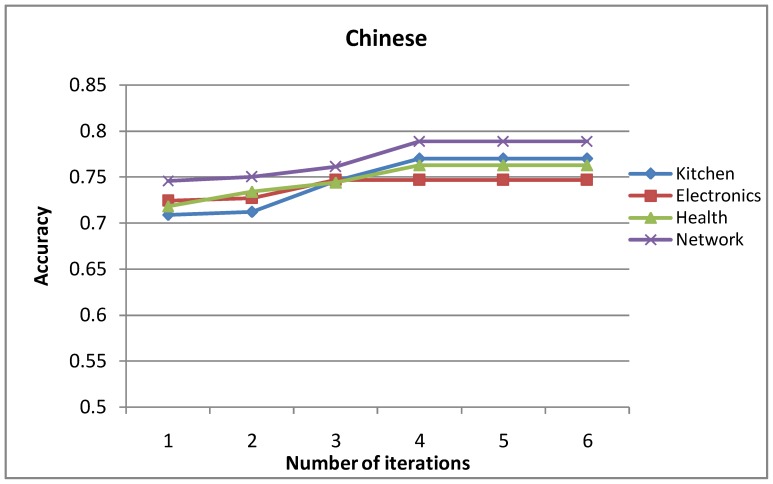
Accuracy of the Chinese opinion lexicon extraction with increasing number of iterations.

Figure 9 and Figure 10 show the accuracy curves of the mutual-reinforcement label propagation algorithm with different numbers of iterations. As seen in Figure 9 and Figure 10, both for English and Chinese, trials on multiple domains show fast convergence of the proposed method.

### Influence of Parameter

**Figure 11:**
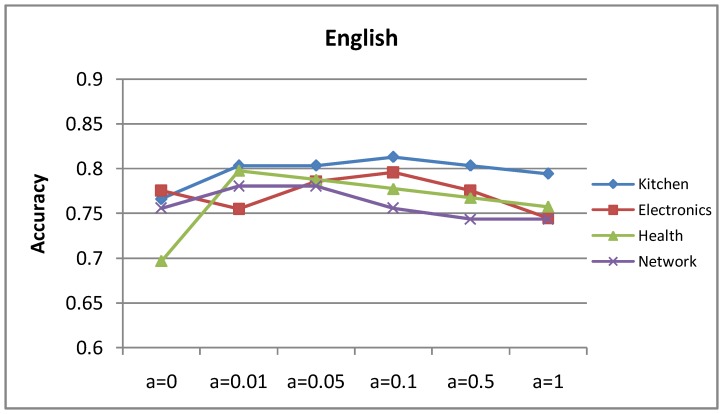
Accuracy of the English opinion lexicon extraction with increasing translation possibility.

**Figure 12:**
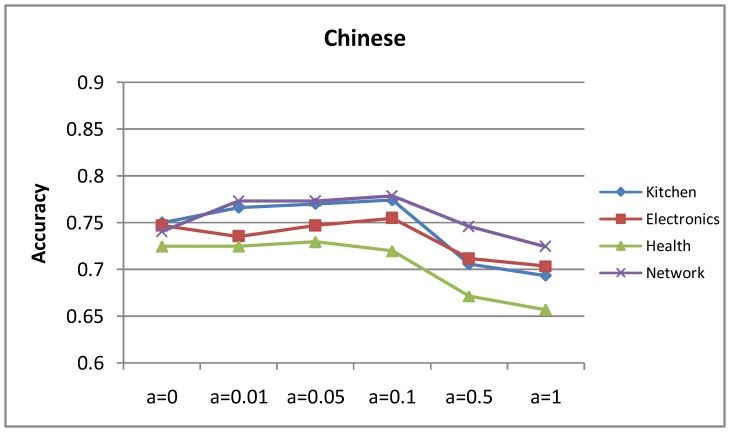
Accuracy of the Chinese opinion lexicon extraction with increasing translation possibility.

Figure 11 and Figure 12 show the accuracy curves of the mutual-reinforcement label propagation algorithm with different parameter 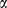. We have tried many different propagating possibilities and finally selected an approximately optimal value, which is 0.1. The selected value is intuitive: if it is too large, the model tends to suffer from translation ambiguity; if it is too small, the bilingual lexicon almost has no effect. More specifically, the translation word in a translation dictionary may be not appropriate for the target word, sometimes even misleading because of translation ambiguity. For example, *cheap* have multiple candidate Chinese translations with different polarities, such as 便宜的 *(low-priced)*, 低档的 *(low-grade)*. The translation might have different polarities from the original intention, for example *cheap* for the hotel price aspect might be translated to 低档的 *(low-grade)*, which is negative. If 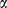 is set to 1 stiffly, this kind of misleading information may be too overwhelming.

## Discussion

In this paper, we propose a cross-language opinion lexicon extraction framework using the mutual-reinforcement label propagation algorithm. Our approach do not use any labeled dataset but instead unlabeled datasets and initial seed words of both languages. The mutual-reinforcement label propagation model is based on bootstrapping. First, for each language, a label propagation algorithm is applied to a large word relation graph, producing a polarity estimate for any given word. Second, a bilingual dictionary is seen as a bridge between two languages to start the bootstrapping process. In order to further improve the performance, we refine the mutual-reinforcement label propagation model from two aspects: seed word selection and graph construction. Experimental results show the effectiveness of our approach. The better performance benefits from the fact that the source language and target language can learn from each other and boost each other through propagating information back and forth.
